# CD44: a key regulator of iron metabolism, redox balance, and therapeutic resistance in cancer stem cells

**DOI:** 10.1093/stmcls/sxaf024

**Published:** 2025-04-22

**Authors:** Taiju Ando, Juntaro Yamasaki, Hideyuki Saya, Osamu Nagano

**Affiliations:** Division of Gene Regulation, Oncology Innovation Center, Research Promotion Headquarters, Fujita Health University, Toyoake 470-1192, Japan; Department of Medical Oncology, Fujita Health University Hospital, Toyoake 470-1192, Japan; Division of Gene Regulation, Oncology Innovation Center, Research Promotion Headquarters, Fujita Health University, Toyoake 470-1192, Japan; Division of Gene Regulation, Oncology Innovation Center, Research Promotion Headquarters, Fujita Health University, Toyoake 470-1192, Japan; Division of Gene Regulation, Oncology Innovation Center, Research Promotion Headquarters, Fujita Health University, Toyoake 470-1192, Japan

**Keywords:** CD44, cancer stem cell, iron metabolism, ferroptosis, SLC7A11

## Abstract

CD44, a multifunctional cell surface protein, has emerged as a pivotal regulator in cancer stem cell (CSC) biology, orchestrating processes such as stemness, metabolic reprogramming, and therapeutic resistance. Recent studies have identified a critical role of CD44 in ferroptosis resistance by stabilizing SLC7A11 (xCT), a key component of the antioxidant defense system, enabling CSCs to evade oxidative stress and sustain tumorigenic potential. Additionally, CD44 regulates intracellular iron metabolism and redox balance, further supporting CSC survival and adaptation to stressful microenvironments. Therapeutic strategies targeting CD44, including ferroptosis inducers and combination therapies, have shown significant potential in preclinical and early clinical settings. Innovations such as CD44-mediated nanocarriers and metabolic inhibitors present novel opportunities to disrupt CSC-associated resistance mechanisms. Furthermore, the dynamic plasticity of CD44 isoforms governed by transcriptional, post-transcriptional, and epigenetic regulation underscores the importance of context-specific therapeutic approaches. This review highlights the multifaceted roles of CD44 in CSC biology, focusing on its contribution to ferroptosis resistance, iron metabolism, and redox regulation. Targeting CD44 offers a promising avenue for overcoming therapeutic resistance and improving the outcomes of refractory cancers. Future studies are needed to refine these strategies and enable their clinical translation.

## Graphical Abstract

**Graphical Abstract:**
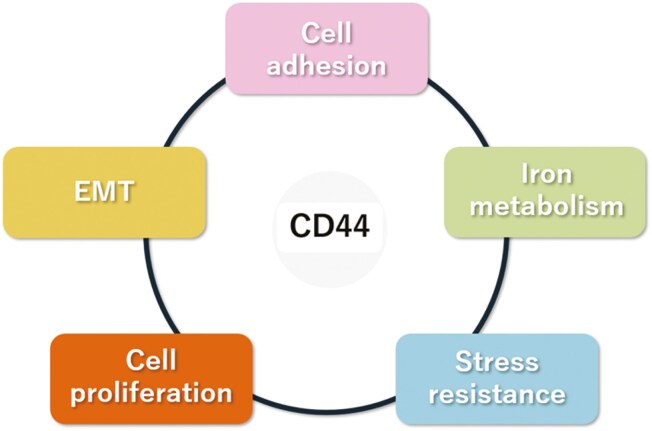

**Significance statement**

Emerging evidence suggests that iron metabolism and redox regulation are intimately linked to cancer stemness and therapeutic resistance. This article highlights a previously underappreciated role of CD44 in orchestrating these processes within cancer stem cells. By integrating recent advances across redox biology, iron homeostasis, and stem cell regulation, we propose a unifying framework in which CD44 acts as a central node in therapy resistance. These insights provide a conceptual basis for future therapeutic strategies targeting the metabolic vulnerabilities of cancer stem cells.

## Introduction

Since Bonnet et al. first identified and isolated cancer stem cells (CSCs) from human acute myeloid leukemia in 1997,^1^ CSCs have attracted significant attention owing to their unique capabilities for self-renewal and differentiation into progeny. CSCs possess a distinct cellular milieu defined by the specific gene expression profiles and unique metabolic pathways, enabling them to withstand various environmental stresses and drive invasion and metastasis into new microenvironments. CSCs can survive cancer drug treatment through mechanisms such as activating drug efflux transporters, enhancing DNA repair, and resisting cell death processes such as apoptosis, thereby posing a significant obstacle to curative therapy.^2^ In recent years, immunotherapy has made significant advances in cancer treatment; however, growing evidence indicates that CSCs possess highly effective immune evasion mechanisms.^3^ Therefore, the effective targeting of CSCs is essential for advancing future therapeutic strategies.

CD44 is widely recognized as a key marker of cancer stem cells (CSCs) and contributes to tumor progression, metastasis, and resistance to therapy. This cell surface protein binds to hyaluronic acid and other ligands, facilitating cell adhesion and activating signaling pathways, including the tyrosine kinase receptor pathways. Beyond its role as a receptor, CD44 functions as a regulator, promoting stemness and protecting tumor cells from stress-induced cell death.^4^ Given its pivotal role in cancer biology, therapeutic strategies targeting CD44-positive cells are being actively developed to improve cancer treatment outcomes.

Increased iron uptake and maintenance of intracellular iron homeostasis are important for maintaining stem cell properties in CSCs, suggesting the existence of unique metabolic pathways that differ from those in non-cancer stem cells (non-CSCs).^5,6^ In addition, recent research has shown that iron uptake is enhanced by the cancer stem cell marker CD44.^7^ Such an increase in iron ions ultimately induces the production of hydroxyl radicals via the Fenton reaction, causing a process called ferroptosis, a form of regulated cell death characterized by iron-dependent lipid peroxidation.^8^ Our previous study showed that CD44 stabilizes SLC7A11(xCT) to enhance antioxidant capacity and inhibit ferroptosis.^9^ Therefore, ferroptosis-inducing therapy may be a promising cancer treatment for treatment-resistant CSCs. In this review, we outline the new functional roles of CD44 in treatment-resistant CSCs that have become clear in recent years, as well as their potential as therapeutic targets.

## Emerging roles OF CD44 IN CSCS

**Figure 1.**
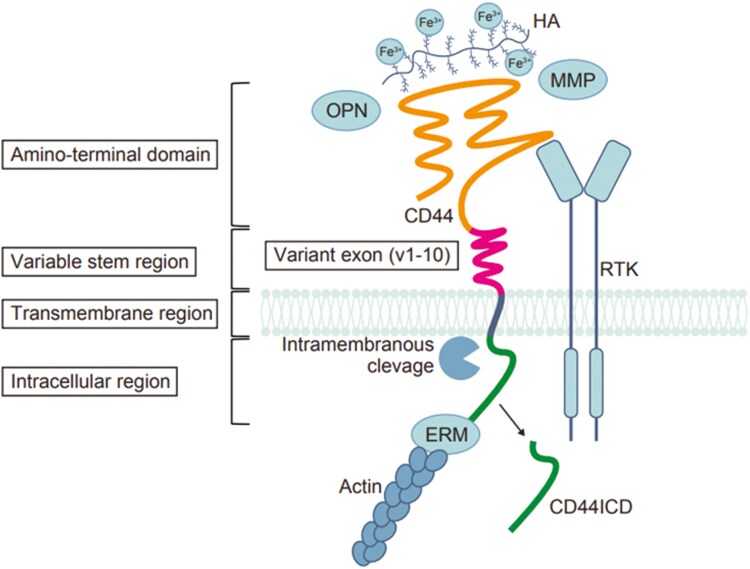
The CD44 protein is located on the cell membrane and consists of four distinct regions: the amino-terminal domain, variable stem region, transmembrane region, and short C-terminal intracellular/cytoplasmic region. CD44 mediates signal transduction through interactions with ligands such as HA and OPN. It also binds to HA-associated iron. Additionally, CD44 functions as a co-receptor in signal transduction via its association with RTKs. Its intracellular region connects to the cytoskeleton through ERM proteins, and upon dissociation, the intracellular domain functions as a transcription factor in the nucleus. Abbreviations: HA, hyaluronic acid; OPN, osteopontin; RTK, receptor tyrosine kinase; ERM, ezrin, radixin, and moesin; CD44ICD, CD44 intracellular domain.

CD44, a cell adhesion molecule that was initially identified in lymphocytes and other immune cells, is widely expressed in vertebrate tissues. In normal epithelia, it acts as a receptor for hyaluronic acid (HA), regulating HA metabolism and supporting adhesion, migration, proliferation, and differentiation.^4,10^ CD44 enhances CSC stemness via its multifunctional role in cancer. Figure 1 shows the structure of CD44 and its function in CSCs.

In 2003, Al-Haji et al discovered that as few as 100 CD44-positive human breast cancer cells can form new tumors.^11^ Accumulating evidence from various types of cancer has led to CD44 being widely recognized as a key molecular marker of CSCs.^12,13^ In addition to its role as an adhesion molecule in cell-matrix interactions, CD44 plays various roles that support the maintenance of stem cell properties. These include interactions with the surrounding microenvironment, not only with hyaluronan but also with other extracellular matrix components such as osteopontin (OPN). CD44 interacts directly with the cytoskeleton via ERM proteins (ezrin, radixin, and moesin) and supports the survival and maintenance of CSCs by engaging tyrosine kinase receptors such as ERBB2 and MET.^14^ Additionally, proteolytic cleavage generates the CD44 intracellular domain (CD44ICD), which participates in intracellular signaling pathways.^15^ CD44-positive CSCs promote tumor growth through pathways such as Wnt/β-catenin and MAPK, while also acquiring therapeutic resistance by upregulating drug efflux pumps such as MDR1 and P-glycoprotein. CD44 signaling activates oxidative stress pathways, induces HIF1α-driven metabolic shifts under hypoxia, and promotes epithelial-mesenchymal transition (EMT), a process in which epithelial cells lose polarity and cell-cell adhesion while acquiring mesenchymal traits, leading to increased motility and invasiveness.^16^ This enhances metastatic potential, plasticity, and stemness in treatment-resistant cancer cells.^14,17,18^

Cancer cells adapt to various environmental stresses through unique metabolic mechanisms. While typical cancer cells rely on the Warburg effect, primarily using glycolysis-a less efficient but rapid ATP production process for glucose metabolism,^19^ CSCs can switch between glycolysis and oxidative phosphorylation (OXPHOS) based on oxygen levels and environmental conditions.^20^ OXPHOS, which occurs in the mitochondria, generates ATP more efficiently by utilizing the tricarboxylic acid cycle and the electron transport chain in an oxygen-dependent manner. CD44 plays a key role in metabolic flexibility by regulating the balance between lactate glycolysis and OXPHOS via pathways involving HIF-1α and AMPKα/mTOR. It modulates the activity of lactate dehydrogenase (LDHA and LDHB) and promotes glycolysis via CD44ICD, which acts as a cotranscription factor.^21^

CD44’s role in metabolism in non-cancerous tissues is becoming clearer. CD44 regulates the glucose and lipid balance and is linked to diabetes and obesity through signaling with ligands such as HA and OPN. In the pancreas, CD44-HA signaling reduces insulin secretion, whereas in the skeletal muscle, it promotes insulin resistance. In the liver, CD44-HA/OPN signaling triggers inflammation, immune cell infiltration, fibrosis, and fatty liver formation. It also increases inflammation and fat accumulation in adipose tissue. CD44-targeted therapies show promise for treating metabolic diseases such as obesity and diabetes, with development currently underway at the preclinical stage.^21^ Thus, CD44 plays a pivotal role as a signaling regulator in maintaining stemness, allowing CD44-positive cells to resist standard drug treatments and drive recurrence and metastasis. Its emerging role as a metabolic regulator has attracted considerable attention as a novel therapeutic target.

CD44 is a single-pass type I transmembrane protein that consists of four regions: the amino-terminal domain, variable stem region, transmembrane region, and short C-terminal intracellular/cytoplasmic region. The *CD44* gene is composed of 20 exons, of which exons 1-5, 16-18, and 20 encode the standard form (CD44s), whereas exons 6-15 (corresponding to variant exons v1–v10) can undergo alternative splicing to alter the stem region conformation, influencing ligand binding and signaling properties, thereby generating functionally distinct various isoforms (CD44v).^22^ In contrast to the widespread expression of CD44s, the expression of CD44v isoforms is limited to specific epithelial cells and certain tumor cells. CD44v has long been associated with various aspects of tumor malignancy.^23-25^ CD44v9 has been reported to be associated with treatment resistance and recurrence rates in gastric cancer. Analyses have revealed that CD44v9-positive tumors are associated with significantly lower recurrence-free survival rates compared to CD44v9-negative tumors, suggesting that CD44v9 may serve as a potential predictive marker for gastric cancer recurrence.^26^ Studies using a transgenic mouse model of gastric cancer (Gan mouse) have demonstrated that CD44v stabilizes SLC7A11, a key factor in resistance to oxidative stress. This stabilization promotes tumor growth, maintenance, and chemoresistance by downregulating p38^MAPK^ and p21^CIP1/WAF^1, which are involved in oxidative stress response and cell differentiation in the stomach.^9^ The functional roles of CD44 isoforms remain controversial. However, CD44s is primarily recognized as a cell adhesion molecule that also activates TGF-β and AKT signaling, promoting EMT and enhancing tumor invasion and metastasis. In contrast, CD44v is mainly considered an activator that regulates various cell membrane proteins, including SLC7A11, to support tumor growth and therapy resistance.^27^

Although CD44v has been considered central to maintaining stemness, recent studies have revealed that in breast cancer, CD44s, but not CD44v, induce stemness via the PDGFRβ/Stat3 signaling cascade regulated by the splicing factor ESRP1, suggesting that CD44v may not be essential for stemness or tumor initiation.^28^ Similarly, CD44s has been positively associated with tumor malignancy in hepatocellular carcinoma, prostate cancer, and gallbladder cancer.^29-31^ In our previous study on metastatic breast cancer cells, we found that ESRP1-mediated upregulation of CD44v promotes lung metastasis.^32^ This apparent contradiction can be reconciled by proposing that CD44s are crucial during the EMT stage at the primary tumor site, whereas CD44v plays a pivotal role in establishing and initiating tumors at metastatic sites.

Interestingly, these findings suggest that the expression of CD44 isoforms may dynamically shift in response to changes in the tumor microenvironment. Recent advances in single-cell analysis and lineage tracing have revealed the plasticity of CSCs, indicating that CSCs and non-CSCs can transition to one another. Consequently, the CSC model has evolved from a rigid hierarchical structure to a more dynamic framework.^33^ This plasticity may partially explain the limited success of CD44-targeted therapies. Therefore, it is crucial to carefully consider the role of CD44 isoforms when designing CD44-targeted therapeutic strategies.

## CD44 and iron metabolism

Iron is an essential trace element for cell proliferation and metabolism. In recent years, various studies have suggested that enhanced iron uptake in CSCs promotes self-renewal, increases metabolic plasticity, and enhances resistance to oxidative stress, highlighting the importance of iron regulatory mechanisms in maintaining cancer stemness.^6^ In normal cells, iron homeostasis is epigenetically regulated by the iron-responsive element/iron-regulatory protein (IRE/IRP) system, which balances iron uptake via the transferrin receptor (TfR) and iron efflux via ferroportin (FPN).^34^ Additional regulatory mechanisms are thought to contribute to iron homeostasis in CSCs. For example, in human melanoma cells, HA-CD44 signaling has been shown to upregulate the expression of TfR on the cell surface, facilitating iron uptake by binding transferrin loaded with Fe³⁺ and mediating its endocytosis.^35^ Interestingly, another CSC marker, CD133, has been shown to play a role in facilitating iron uptake by human colorectal cancer cells.^36^ Recent studies have shown that CD44 directly facilitates intracellular iron uptake by mediating endocytosis of iron-bound HA. Additionally, iron-dependent histone methylation regulates the expression of EMT-related genes, including CD44, CD109, VIM, and FN1 (which encode vimentin and fibronectin), by promoting the demethylation of H3K9me2.^7^ This iron-dependent epigenetic regulation via CD44 is crucial as it enhances the expression of genes that sustain CSC characteristics. Unlike the conventional expression of TfR, which is downregulated by excess iron through the IRE/IRP system, CD44 operates via a positive feedback loop that amplifies its activity in response to iron imports. This mechanism enables CSCs to survive and thrive in iron-deficient or high-stress environments, where traditional iron-uptake pathways are suppressed due to elevated intracellular iron levels. Interestingly, prolactin, a lactation hormone secreted by the pituitary gland, may influence CD44-mediated iron uptake,^37^ suggesting a potential hormonal influence on CSC iron metabolism. These findings underscore CD44’s pivotal role in iron regulation, and suggest that it is a promising target for novel therapies designed to disrupt CSC iron metabolism, overcome treatment resistance, and achieve curative outcomes.

## CD44-mediated evasion of ferroptosis

**Figure 2.**
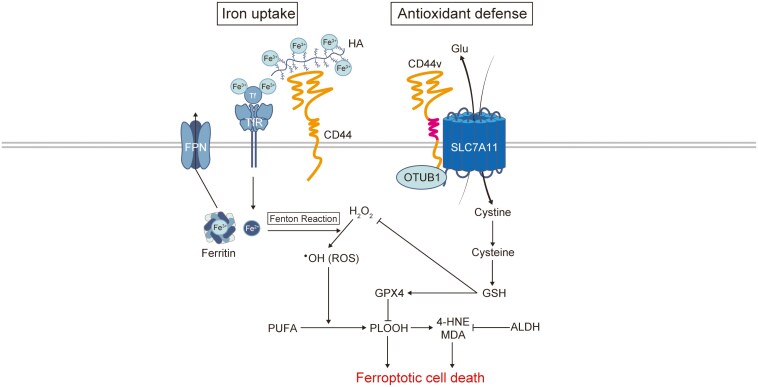
The mechanism of intracellular iron and ferroptosis regulation by CD44. Iron is imported into cells via two primary pathways: the transferrin receptor (TfR)-mediated pathway and the CD44-mediated pathway. The latter involves binding to hyaluronic acid. Once internalized, iron is either stored in ferritin or exported via ferroportin (FPN). However, some iron participates in Fenton reactions, converting H₂O₂ into reactive oxygen species (ROS). These ROS induce lipid peroxidation of polyunsaturated fatty acids (PUFAs), leading to the formation of lipid hydroperoxides (PLOOH) and their decomposition products, 4-hydroxynonenal (4-HNE) and malondialdehyde (MDA). The accumulation of these products triggers ferroptosis. Detoxification of lipid peroxidation products is mediated by aldehyde dehydrogenase (ALDH). The cell membrane protein SLC7A11(xCT) is stabilized by CD44 and OTUB1, thereby facilitating the exchange of extracellular cystine with intracellular glutamate (Glu). The imported cystine is then converted into cysteine, which helps produce GSH. GSH not only eliminates H_2_O_2_ through its reducing activity, but it also removes PLOOH as a cofactor of GPX4. Abbreviations: CD44v, CD44 variant; •OH, hydroxyl radical;.

Ferroptosis, a form of iron-dependent programmed cell death characterized by lipid hydroperoxide accumulation, plays a key role in suppressing tumor growth and overcoming resistance to cancer therapies. Cancer cells activate antioxidant defenses to evade ferroptosis. Figure 2 illustrates the regulation of iron metabolism and ferroptosis, and highlights the central role of CD44. The SLC7A11/GPX4 axis is a critical defense mechanism against ferroptosis.^38^ The cystine/glutamate antiporter System Xc-, located

on the cell membrane, consists of two subunits: SLC7A11 (xCT) and SLC3A2 (CD98). This system exchanges extracellular cystine with intracellular glutamate (Glu). Once inside the cell, cystine is converted into cysteine, which helps produce glutathione (GSH). GPX4, an enzyme found in both the cytoplasm and mitochondria, uses GSH to neutralize hydrogen peroxide, thereby protecting cells from oxidative damage.^39,40^ Ferroptosis is triggered by the accumulation of phospholipid hydroperoxides (PLOOHs) that are formed during the lipid peroxidation of polyunsaturated fatty acids (PUFAs). GPX4, using GSH as a cofactor, reduces PLOOHs, prevents their accumulation, thereby inhibiting ferroptosis.^41^

In 2006, CD44-positive cells isolated from human breast cancer cells, known as mammospheres, showed both radiation resistance and low levels of reactive oxygen species (ROS).^42^ In 2009, studies using MMTV-Wnt-1 mice demonstrated that CD44-positive CSCs have reduced ROS levels owing to the increased expression of antioxidant defense genes, including those involved in GSH synthesis.^43^ These findings suggest that CD44-positive CSCs protect themselves from oxidative stress by enhancing their antioxidant capacities. Additionally, we confirmed that CD44v increases intracellular GSH levels in cancer cells.^9^

A stable supply of cystine is essential for the synthesis of GSH; therefore, we focused on System Xc- and found that CD44v stabilizes SLC7A11 on the cell membrane, boosting intracellular GSH levels and reducing ROS levels.^9^ Recent studies have shed light on the mechanism by which CD44 regulates the expression of SLC7A11. OTUB1, a deubiquitinase involved in cancer progression and immune responses, binds to the N-terminus of SLC7A11, preventing its degradation and stabilizing it on the cell membrane.^44^ CD44 binds to the C-terminus of SLC7A11, enhancing its interaction with OTUB1. This interaction promotes the stabilization and expression of SLC7A11, thereby supporting ferroptosis resistance and tumor growth, emphasizing the critical role of the CD44/OTUB1-SLC7A11 pathway.

Long noncoding RNAs (lncRNAs), non-protein-coding transcripts over 200 nucleotides in length, are crucial regulators of the gene expression, chromatin structure, and cellular signaling, and play significant roles in development, cell differentiation, and disease progression.^45^ Recent studies suggest that certain lncRNAs regulate the CD44/OTUB1/SLC7A11 pathway. The lncRNA HOTAIRM1 modulates CD44 alternative splicing by inhibiting m6A methylation of CD44 precursor transcripts through the m6A demethylase FTO. This prevents recognition by the m6A reader protein YTHDC1, leading to increased CD44v expression.^46^ Additionally, FTO has been reported to enhance ferroptosis resistance by reducing m6A methylation of OTUB1 precursor transcripts, thereby upregulating OTUB1 expression.^47^ Another lncRNA, CTC-490G23.3, is suggested to regulate CD44 alternative splicing via the RNA-binding protein PTBP1, promoting CD44v expression.^48^ Understanding the regulatory mechanisms of the CD44/OTUB1/SLC7A11 pathway may be crucial for developing more effective therapies.

A regulatory pathway involving CD44/SLC7A11 mediated by the tumor suppressor p53 has been reported, wherein p53 directly binds to the SLC7A11 promoter, suppressing its transcription and downregulating its expression.^49^ Additionally, p53 suppresses the expression of CD44 by binding to its promoter.^50^ p53 increases the ferroptosis sensitivity of CD44-positive cells by suppressing the expression of CD44 and SLC7A11. Recent studies have shown that the p53-induced RNA-binding protein ZMAT3 regulates CD44 splicing, reducing CD44v expression while maintaining CD44s expression.^51^ Knockdown of ZMAT3 using siRNAs in human colon cancer cells significantly increased cell viability and clonogenicity. In contrast, knockdown of CD44v alone or CD44v and ZMAT3 simultaneously dramatically reduced cell viability and clonogenicity. Given that the plasticity of CD44 isoforms plays a crucial role in cancer stem cell adaptation, ZMAT3-mediated regulation of CD44 splicing may shift this balance, ultimately suppressing CSC properties. Further research on the p53-mediated regulation of the CD44/SLC7A11 pathway is needed.

## Therapeutic approaches targeting CD44 in CSC metabolism

**Figure 3.**
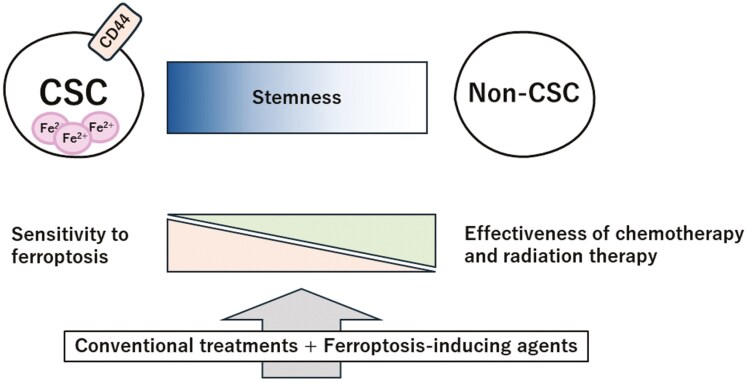
The concept of combination therapy. CSCs are less sensitive to chemotherapy and radiotherapy than non-CSCs but have a higher dependency on iron, rendering them more vulnerable to ferroptosis. Combining conventional treatments with ferroptosis-inducing agents targets both mechanisms simultaneously, aiming to maximize therapeutic efficacy. Abbreviations: CSC, cancer stem cell; non-CSC, non-cancer stem cell.

Inducing ferroptosis in treatment-resistant CSCs is a promising strategy to prevent recurrence and metastasis. We previously showed that the inhibition of SLC7A11 with sulfasalazine, a drug used for rheumatoid arthritis and ulcerative colitis, induced ROS production and restored treatment sensitivity in CD44v-positive cells.^9,52^ In addition, both in vitro and in vivo studies have shown that ferroptosis-inducing agents can restore treatment sensitivity in cancers resistant to anti-epidermal growth factor receptor (EGFR) and anti-vascular endothelial growth factor receptor (VEGFR) therapies.^53^ It has previously been shown that sulfasalazine exhibits limited efficacy in CD44-negative non-CSCs.^54^ Although this is favorable from the perspective of reducing treatment toxicity, considering the plasticity of CSCs, treatments targeting only ferroptosis-sensitive stem cells may be insufficiently effective. Therefore, a combination of conventional therapies with ferroptosis-inducing agents is considered a more effective approach (**Fig. 3**). For instance, we previously reported that the combination of cisplatin and sulfasalazine exerted a synergistic antitumor effect, surpassing the efficacy of each individual agent.^9^ Similarly, favorable outcomes have been observed in both in vitro and in vivo studies when ferroptosis-inducing agents were combined with clinically used anticancer drugs such as paclitaxel, olaparib, cetuximab, and gefitinib.^53,55^

Clinical trials exploring treatments using ferroptosis inducers are currently underway. In a phase I trial involving patients with advanced gastric cancer, sulfasalazine treatment effectively reduced the proportion of CD44v-positive cancer cells and decreased intratumoral GSH levels, demonstrating its functionality in humans.^56^ Several clinical trials of combination therapies have also been conducted. In a phase I dose-escalation study of cisplatin combined with sulfasalazine in patients with advanced gastric cancer resistant to cisplatin, combination therapy was found to be manageable.^57^ Although factors such as suboptimal sulfasalazine dosing and patient background may have limited its therapeutic effect, one patient achieved stable disease for over 4 months, In a phase I trial combining sulfasalazine with cisplatin and pemetrexed for advanced non-small cell lung cancer, the addition of sulfasalazine showed a response rate and progression-free survival (PFS) comparable to monotherapy.^58^ Further clinical trials are warranted to evaluate the efficacy of combining conventional treatments with induction of ferroptosis.

Cancer cells often maintain ROS defense even when SLC7A11 is inhibited, limiting the effectiveness of ferroptosis inducers as monotherapy. Through drug screening, we identified dyclonine, an oral anesthetic and ALDH enzyme inhibitor, as a compound that re-sensitizes sulfasalazine-resistant cancer cells to SLC7A11 inhibitors.^59^ Dyclonine enhances SLC7A11-targeted therapy and induces necrotic cell death in squamous cell carcinoma. In a syngeneic gastric cancer model, a combination therapy with dyclonine and sulfasalazine showed significant efficacy. Oxyfedrine, a structural analog of dyclonine, enhances the effects of sulfasalazine and offers a safer therapeutic option, making it a promising candidate for clinical use.^60^ The combination of ferroptosis inducers and sensitizing agents is a promising strategy for cancer treatment.

Recently, treatments using nanocarriers that leverage CD44’s affinity for HA have been developed and strategies for inducing selective ferroptosis at the cellular level have been actively explored.^61^ For example, iron-platinum alloy nanoparticles modified with HA have been shown to overcome therapy resistance by inducing ferroptosis in EMT-state CD44-positive lung cancer cells via CD44-HA-mediated endocytosis.^62^ Similar studies are also underway on bladder cancer, gastric cancer, hepatocellular carcinoma, and breast cancer.^63-66^ Therefore, combining targeted chemotherapy with ferroptosis inducers is being explored to achieve synergistic effects. Using CD44-mediated nanocarriers to deliver both agents is expected to enhance tumor suppression while minimizing toxicity.^67,68^

Although challenges remain in its clinical application, combination therapies with ferroptosis inducers show great promise. Identifying biomarkers for treatment sensitivity is crucial, considering ferroptosis sensitivity across different tumor types and microenvironments as well as the context-dependent effects of CD44 on stemness. Our previous study identified glutamine metabolism (glutaminolysis)-related genes as key determinants of sulfasalazine sensitivity in CD44-positive CSCs,^69^ highlighting their potential as markers for the efficacy of ferroptosis-inducing therapies.

## Summary

CD44 plays a pivotal role in cancer biology, and is a key regulator of CSC characteristics and metabolic pathways. Its involvement in ferroptosis resistance, particularly through the stabilization of SLC7A11, highlights the therapeutic potential of targeting the CD44/SLC7A11 axis to overcome treatment resistance. The complexity of CD44/SLC7A11 regulation, which is influenced by factors such as deubiquitinase OTUB1 and context-dependent isoform plasticity, underscores the need for nuanced therapeutic approaches.

CD44’s dual role in redox control and intracellular iron regulation further emphasizes its importance as both a CSC marker and a redox regulator. Integrating the induction of ferroptosis with conventional therapies can eliminate CSCs and achieve curative outcomes. Additionally, targeting CD44’s broader involvement in CSC metabolism and unique metabolic pathways offers new therapeutic avenues. A deeper understanding of the role of CD44 in CSC biology and ferroptosis resistance is essential to develop innovative strategies to combat treatment-resistant cancers.

## Data Availability

Not applicable. This review article is based entirely on previously published literature, all of which is appropriately cited within the text.
